# Experimental Directory Structure (Exdir): An Alternative to HDF5 Without Introducing a New File Format

**DOI:** 10.3389/fninf.2018.00016

**Published:** 2018-04-13

**Authors:** Svenn-Arne Dragly, Milad Hobbi Mobarhan, Mikkel E. Lepperød, Simen Tennøe, Marianne Fyhn, Torkel Hafting, Anders Malthe-Sørenssen

**Affiliations:** ^1^Centre for Integrative Neuroplasticity, University of Oslo, Oslo, Norway; ^2^Department of Physics, University of Oslo, Oslo, Norway; ^3^Department of Biosciences, University of Oslo, Oslo, Norway; ^4^Institute of Basic Medical Sciences, University of Oslo, Oslo, Norway; ^5^Department of Informatics, University of Oslo, Oslo, Norway

**Keywords:** file format, data storage, data management, analysis, Python

## Abstract

Natural sciences generate an increasing amount of data in a wide range of formats developed by different research groups and commercial companies. At the same time there is a growing desire to share data along with publications in order to enable reproducible research. Open formats have publicly available specifications which facilitate data sharing and reproducible research. Hierarchical Data Format 5 (HDF5) is a popular open format widely used in neuroscience, often as a foundation for other, more specialized formats. However, drawbacks related to HDF5's complex specification have initiated a discussion for an improved replacement. We propose a novel alternative, the Experimental Directory Structure (Exdir), an open specification for data storage in experimental pipelines which amends drawbacks associated with HDF5 while retaining its advantages. HDF5 stores data and metadata in a hierarchy within a complex binary file which, among other things, is not human-readable, not optimal for version control systems, and lacks support for easy access to raw data from external applications. Exdir, on the other hand, uses file system directories to represent the hierarchy, with metadata stored in human-readable YAML files, datasets stored in binary NumPy files, and raw data stored directly in subdirectories. Furthermore, storing data in multiple files makes it easier to track for version control systems. Exdir is not a file format in itself, but a specification for organizing files in a directory structure. Exdir uses the same abstractions as HDF5 and is compatible with the HDF5 Abstract Data Model. Several research groups are already using data stored in a directory hierarchy as an alternative to HDF5, but no common standard exists. This complicates and limits the opportunity for data sharing and development of common tools for reading, writing, and analyzing data. Exdir facilitates improved data storage, data sharing, reproducible research, and novel insight from interdisciplinary collaboration. With the publication of Exdir, we invite the scientific community to join the development to create an open specification that will serve as many needs as possible and as a foundation for open access to and exchange of data.

## Significance statement

An alternative storage solution that improves on certain drawbacks of Hierarchical Data Format 5 (HDF5) is to use directories in the file system to define a hierarchy, and store data in binary files, and metadata in text files. While this strategy can be deployed in various ways by research groups, no common standard for such a storage solution exists. Experimental Directory Structure (Exdir) is a proposal to standardize this storage solution. We envision the establishment of such a standard and present Exdir to the community as a starting point.

## 1. Introduction

Technology development is continuously driving science to new discoveries. In neuroscience, advancements in genetic tools, recording technology, and computer power have paved the avenue to reveal the underlyings of the healthy and diseased brain. Modern neuroscience usually involves recordings and perturbation at many levels, generating a range of data including imaging, electrophysiology, behaviors, perturbations, and molecular biology. Publication of raw data is acknowledged as critical to enable reproducible research and global large-scale collaborative projects and metadata analyses (Nelson, 2009). However, data from different acquisition systems come in a multitude of data formats that need to be readable for all relevant analysis software and stored for long-term archival. Acquisition systems often use proprietary and specialized formats tailored to data produced by specific types of equipment or software. However, these specialized formats have little applicability outside their intended purpose, making them inaccessible for extended use. In contrast, general-purpose formats can store data for multiple types of equipment and software. When based on open standards, general-purpose formats facilitate data sharing.

Hierarchical Data Format 5 (HDF5) (The HDF Group, 1997-2018) is a popular and open general-purpose format capable of storing many large and annotated datasets in a hierarchical structure within a single file. HDF5 is the basis of many formats in neuroscience, including the recent collaborative format, Neurodata Without Borders (NWB) (Teeters et al., 2015). However, issues with HDF5 have recently surfaced in the neuroscience community (Rossant, 2016b). Many of these are due to the complex specification of HDF5 and its use of a single binary file to store all the data. The metadata is not human-readable and the binary format is not optimal for version control systems. Further, the use of a single file increases the severity of data corruption, because corruption in a single dataset can affect the entire file. Additionally, while it is possible to store raw data as a sequence of bytes with the HDF5 opaque datatype, it is inconvenient to open the raw data in external applications. These issues have sparked a discussion in the wider scientific community on whether HDF5 should be replaced by alternative data formats or if its large feature set outweighs the disadvantages (Hinsen, 2016).

Here, we propose a novel specification, Experimental Directory Structure (Exdir) as an alternative that circumvents the drawbacks of HDF5 and takes advantage of existing, open data formats. Exdir follows the abstract data model used in HDF5^1^, but stores data and metadata in directories to avoid the vulnerability and rigidity associated with storing all data in a single file. Datasets are stored in binary NumPy (van der Walt et al., 2011) files, while attributes and metadata are stored in YAML (Ben-Kiki et al., 2009) text files. Raw data, such as images and time series obtained during data acquisition, can also be stored in Exdir alongside the binary NumPy files. This allows raw data to be organized inside an Exdir hierarchy without any prior conversion, even when the data is composed of multiple formats.

The full name (Experimental Directory Structure) reflects that Exdir started out with a goal to efficiently store experimental data alongside analyzed data (and that the specification initially was in rapid development in our lab). However, there is no limitation to the type of data that can be stored in Exdir. Any type of data that can be stored in an HDF5 dataset should be possible to store in an Exdir dataset, and other types of data can be stored as raw data within the hierarchy.

Exdir is ready to use with a reference implementation in Python, a command-line client, and a graphical browser. The application programming interface (API) of the reference implementation is compatible with h5py (Collette, 2013), a popular HDF5 library for Python. The code is open source and hosted on GitHub^2^.

The idea of an HDF5-replacement based on a hierarchy of directories is already present in the scientific community (Rossant, 2016a), but to the best of our knowledge no formal specification has been introduced. The lack of such of a specification limits collaboration through data sharing, and inhibits development of analysis tools. Exdir represents the introduction of a specification that enables novel insight from interdisciplinary collaboration by facilitating reproducible research through improved data storage and sharing. With the publication of Exdir, we invite the scientific community to join the development to create an open specification that will serve as many needs as possible.

## 2. Existing alternatives

### 2.1. Hierarchical data format (HDF5)

The HDF5 format holds many advantages over alternative data formats (see e.g., Teeters et al., 2015). However, the HDF5 format also has crucial disadvantages, such as described in Greenfield et al. (2015). In the list below, we have summarized the limitations and challenges from Greenfield et al. (2015) that are most relevant for scientific use along with some additional drawbacks which are addressed with Exdir:

1. Metadata is stored in a binary format which makes it unreadable without tools that read HDF5 files. This also makes the metadata unavailable for text-based command line tools.
2. The specification for HDF5 files is large and complex and there is only one de-facto implementation of HDF5 in C that most HDF5-libraries use. Because of the complex specification, this implementation is hard to improve by external contributors. Furthermore, the dependency on one large implementation makes it hard to write software which reads and writes HDF5 files in ways that have not been anticipated by the implementation developers.
3. Like all data formats, HDF5 files are susceptible to data corruption. However, because HDF5 stores all data and metadata in a single file, data corruption in one part of an HDF5 file has a chance of corrupting the entire file.
4. Attributes in HDF5 do not support deeply nested structures, like JSON data, YAML data, or Python dictionaries.
5. External version control systems such as Git^3^ and incremental backup systems do not work optimally with HDF5 files because all datasets and metadata are stored in a single binary file. This makes it appear as if the entire file has changed when changes are made to a single dataset.
6. Comparing files in binary formats like HDF5 requires specialized tools. However, text-based formats have a wide range of tools that allow line-by-line comparisons, such as *diff* (MacKenzie et al., 2015), and *wdiff* ^4^, or the graphical tools *meld*^5^ and *kdiff3* (Eibl, 2002–2007).
7. Deleting datasets in HDF5 files only removes a reference to the data, while the data remains on disk (except if the dataset is the last remaining object in a page allocated at the end of the file)^6^.
8. Raw data from acquisition or analysis is hard to access from external applications when stored inside an HDF5 file. An alternative is to organize raw data in a separate hierarchy outside the HDF5 file, but this makes the raw data detached from the internal hierarchy and inconvenient to annotate.

### 2.2. Other formats

Greenfield et al. (2015) propose a new format (Advanced Scientific Data Format, ASDF) to address many of the above mentioned problems. Similar to Exdir, ASDF also embraces YAML for metadata, but it also stores and organizes binary data in the same YAML file. Storing the data in one file has the same increased risk of data corruption as HDF5 and makes it harder for version control systems to keep track of incremental changes. Furthermore, ASDF does not provide a convenient way to store raw data in the internal hierarchy.

Some specifications, such as the Brain Imaging Data Structure (BIDS) (Gorgolewski et al., 2016), also approach the above problems by using the file systems to define the data hierarchy, which is similar to the solution we propose with Exdir. However, these specifications often serve only the purpose of one or few particular scientific fields, such as neuroscience.

Exdir is not restricted to data from one scientific field and could be used as an alternative where the flexibility of HDF5 is currently enjoyed. Because Exdir has the same abstract data model as HDF5, it should be fairly easy to transition from HDF5 to Exdir for formats based on HDF5 also in other fields, such as geosciences (NetCDF4, Rew et al., 2006; Unidata, 2018), medical imaging (MINC, Vincent et al., 2004), and neutron, X-ray and muon science (NeXus, Könnecke et al., 2015)^7^.

In Table 1, some of the commonly used open formats in neuroscience are listed. Some of these formats are discussed by Teeters et al. (2015) where they also introduce Neurodata Without Borders (NWB), a format recently developed in an attempt to unify cellular-based neurophysiology data and break down barriers for data sharing. Many of these formats, including NWB, are based on HDF5 and therefore share the same advantages and disadvantages as HDF5. Because Exdir is compatible with the abstract data model of HDF5, these formats could be based on Exdir in the future.

**Table 1:** Overview of commonly used open formats in neuroscience.

**Name**	**HDF5**	**Notes**	**References**
NWB	Yes		Teeters et al., 2015
Kwik	Yes		Kadir et al., 2014; Rossant et al., 2015
BRAINformat	Yes		Rübel et al., 2015
Open Ephys	Yes/No	Binary format specifically designed for electrophysiological data. HDF5 optional.	Siegle et al., 2015
NeuroShare	No	API to access binary formats and a binary format specifically designed for electrophysiological data.	neuroshare.org
Neo	N/A	In-memory data format for Python. Uses different formats for file storage.	Garcia et al., 2014
CARMEN NDF	(Yes)	Specifically designed for neuroscience. Stores hierarchical structure and metadata in XML files. Data is stored in MATLAB .mat files, which are technically HDF5 files.	carmen.org.uk
Nix	Yes	Adds a layer on top of the abstract data model that standardizes annotation of data. Directory-based backend in development.	Stoewer et al., 2014
odML	No	Only applies to metadata.	Grewe et al., 2011
NSDF	Yes	Format for neuroscience simulation data	Ray et al., 2016

### 2.3. Requirements of a new specification

We share many of the requirements reviewed in detail by Greenfield et al. (2015) for the ASDF format. To meet the challenges, a data format should:

1. Have an intrinsic hierarchical structure.
2. Be human-readable.
3. Be based on existing standards.
4. Be easy to extend.
5. Have efficient mechanisms to update data.
6. Have support for both text and binary data.

In addition to the above mentioned requirements, we want Exdir to:

7. Minimize the risks and consequences of data corruption.
8. Have a simple, yet flexible specification.
9. Be flexible to data modifications.
10. Be easy to use in ways that have not been anticipated by the authors.
11. Be based on the same abstractions as HDF5 to make it easy to port HDF5-based solutions.
12. Provide a convenient way to store raw data in the same hierarchy.

None of the existing formats known to the authors fulfill all of the mentioned requirements.

## 3. Standards used in exdir

To fulfill the requirements stated in section 2.3, we propose a new specification, Exdir, which is based on a standardized directory structure and established open-source file formats. The structure follows the abstract data model used in HDF5, but Exdir uses regular file system directories to define the object hierarchy, and stores datasets, attributes, and corresponding metadata in separate files.

Exdir uses YAML files to store metadata and attributes. YAML is a human-readable and human-writable format that supports data types such as strings, numbers, lists, and key-value pairs. Furthermore, libraries for YAML support exist for most major programming languages, including Python, C/C++, Java, Rust, and MATLAB^8^^,^^9^.

YAML files in Exdir are based on version 1.2 of the YAML specification^10^, but with some additional restrictions that are added because not all features of the full YAML specification are necessary for storing attributes and metadata in Exdir. The restrictions are made to make the format simpler and easier to parse for humans, which we believe improves data sharing. Further, the format should also be easier to parse programmatically, which could open up for the implementation of more efficient parsers in the future. Although some of the restrictions may be removed in a future version of Exdir, we want to start out with a strict subset of YAML and extend only when a clear need is identified for more advanced features. The restricted subset of YAML used in Exdir is compatible with the full YAML 1.2 specification.

The restrictions added to attribute and metadata files in Exdir are listed below. References to individual sections of the YAML 1.2 specification are shown in parentheses:

1. Only tags from the Failsafe, JSON, and Core schemas (sections 10.1–10.3) are allowed, which means that the supported types in YAML files in Exdir are: map, sequence, string, null, boolean, integer, and floating point.
2. Directives must not be used (section 6.8).
3. Node properties must not be used (section 6.9), which also means that explicit or application-specific tags must not be used and anchors must not be used.
4. Complex mapping keys must not be used (sections 2.2 and 8.2.2), which also means that the complex mapping key indicator, “?” is not allowed.
5. String values must not be used in plain style (section 7.3.3), which also means that string values must be enclosed in double or single quotes. However, keys are encouraged to be in plain style (more on key naming convention below).
6. Flow style must not be used for maps or sequences (section 7.4), which means that curly or square braces must not appear outside of string values.
7. Empty keys must not be used (section 7.4)
8. Block scalar styles must not be used (section 8.1), which also means that multi-line strings should be enclosed in double or single quotes.

Writing files with these restrictions may not always be easily done with existing YAML libraries in all programming languages. We have therefore chosen to read files with the full YAML 1.2 specification in our Python reference implementation and will only issue warnings whenever we encounter YAML files that do not adhere to the above restrictions. We recommend developers of Exdir libraries in other languages to do the same. The intention of the restrictions is not to introduce yet another file format, which could limit the adoption of Exdir, but to ease the transition to a stricter subset of YAML that is easier to parse by both humans and computers.

We also recommend users to adhere to a strict naming convention for keys:

1. Keys should only contain ASCII letters (a–z and A–Z), numbers (0–9), underscores (_) and hyphens (-).
2. Keys should not be surrounded by quotation marks.

This is not part of the Exdir specification because it would conflict with the more relaxed naming rules for keys in HDF5, which in turn could complicate a transition from HDF5 to Exdir in projects where such keys are used. If the first recommendation is broken and custom characters are introduced, we recommend also to break the second rule and add quotes around the key name in question.

JSON was also considered as a format for metadata and attributes, but was rejected because it requires additional tokens such as curly braces to delimit objects and commas to separate key-value maps. While JSON files are human-readable and widely supported, we find the additional tokens to make the files harder to write and maintain manually. The additional tokens play an important role when JSON is used as a serialization format to stream data over network protocols, where they are used to verify that all objects are complete and that the transmission was not interrupted. This is however of limited use in Exdir because we are primarily concerned with creating, editing, storing, and transferring data in bulk, rather than streaming serialized data.

It can be interesting to note that one of the improvements in YAML 1.2 over YAML 1.1 was to make YAML a superset of JSON. However, by introducing our above restrictions, we essentially end up with a subset of YAML that is no longer compatible with JSON. This is a consequence of our emphasis on human readability and simplicity over compatibility with JSON. Further, our restrictions also remove YAML features used to represent arbitrary native data structures, such as explicit tags. This is something we believe is necessary to improve the readability of the attribute files in Exdir. To store complex data structures, we encourage the use of human-readable structures in the attributes. For instance, physical quantities can be stored as a map with two key-value pairs: one for the unit and one for the magnitude. Conversion to and from in-memory objects can be done using Exdir plugins (section 5.2). For very complex data structures or binary data, we encourage users to store this in a dataset or as raw data.

Exdir uses the NumPy format^11^ to store datasets as binary data. This is a simple, efficient, and widely used file format. Furthermore, there exist libraries to load NumPy files in several languages such as Matlab^12^, Rust (Potocek et al., 2018), R (Eddelbuettel and Wu, 2016), and C/C++^13^.

The hierarchical structure of Exdir benefits from the hierarchy of directories in file systems. It is an existing specification which is familiar to computer users. By using this inherent hierarchy, Exdir makes it possible for a user to browse any Exdir object with a native file explorer. Further, the use of regular directories allows raw data from acquisition to be stored and accessed in the same hierarchy and annotated together with the rest of the data.

Parallel reading and writing to separate objects (such as two different Group objects or Dataset objects) in an Exdir directory is not a problem since they are separate files and directories. Parallel operations on separate files are handled by the operating system. This is in contrast to HDF5, where parallel read/write operations must be handled by the HDF5 library because all objects are stored in the same file. Parallel reading and writing to a single Exdir object is on the other hand currently not supported in the reference implementation. Objects are also not locked when opened for reading or writing. The user must currently take care to assure that two processes do not modify the same objects simultaneously. Support for locking and parallel read/write operations to the same objects is planned for a future version of Exdir.

As each dataset is stored in its own directory, the risk of data corruption is reduced. If one dataset is corrupted, it is unlikely to affect the other files in a directory. This separation also makes Exdir avoid the problem of data remaining after deletion in HDF5 and taking up space. Deleting a dataset in Exdir immediately frees up disk space.

When accessing large Exdir File objects, one can easily retrieve and share subtrees of the main hierarchy by copying the corresponding directories. This reduces memory, CPU, and server-communication costs by keeping the size of data handled to a minimum. When sharing Exdir data with others, one can use readily available compression file formats such as .zip or .tar.gz. Alternatively, the Exdir file can be converted into a HDF5 file, which can be used to exchange data with others (see section 6.3).

In the future, it could be possible to extend the Exdir specification to support additional standard data formats in addition to the NumPy format. It will for instance be of interest to add support for tabular data with named columns, such as CSV. This has however been postponed to a future version because such a format needs to be carefully evaluated based on interoperability, numerical precision, and more. We would also like to receive feedback from the wider scientific community about their needs for storing tabular data before reaching a conclusion.

In order to maintain the simplicity of Exdir and the reference implementation, data consistency verification is not built into Exdir. We envision the use of dedicated software for versioning and consistency, such as git^14^ and git-annex^15^. For instance, plugins (see section 5.2) can be developed to use version control systems like git to track each object and their checksums in an Exdir directory. This will make it possible to detect when files have changed independently. This also allows Exdir to be combined with git-based systems like GIN, which are tailored toward cloud-based handling of large datasets (Garbers et al., 2017).

## 4. Basic structure of exdir directories

**Figure 1:**
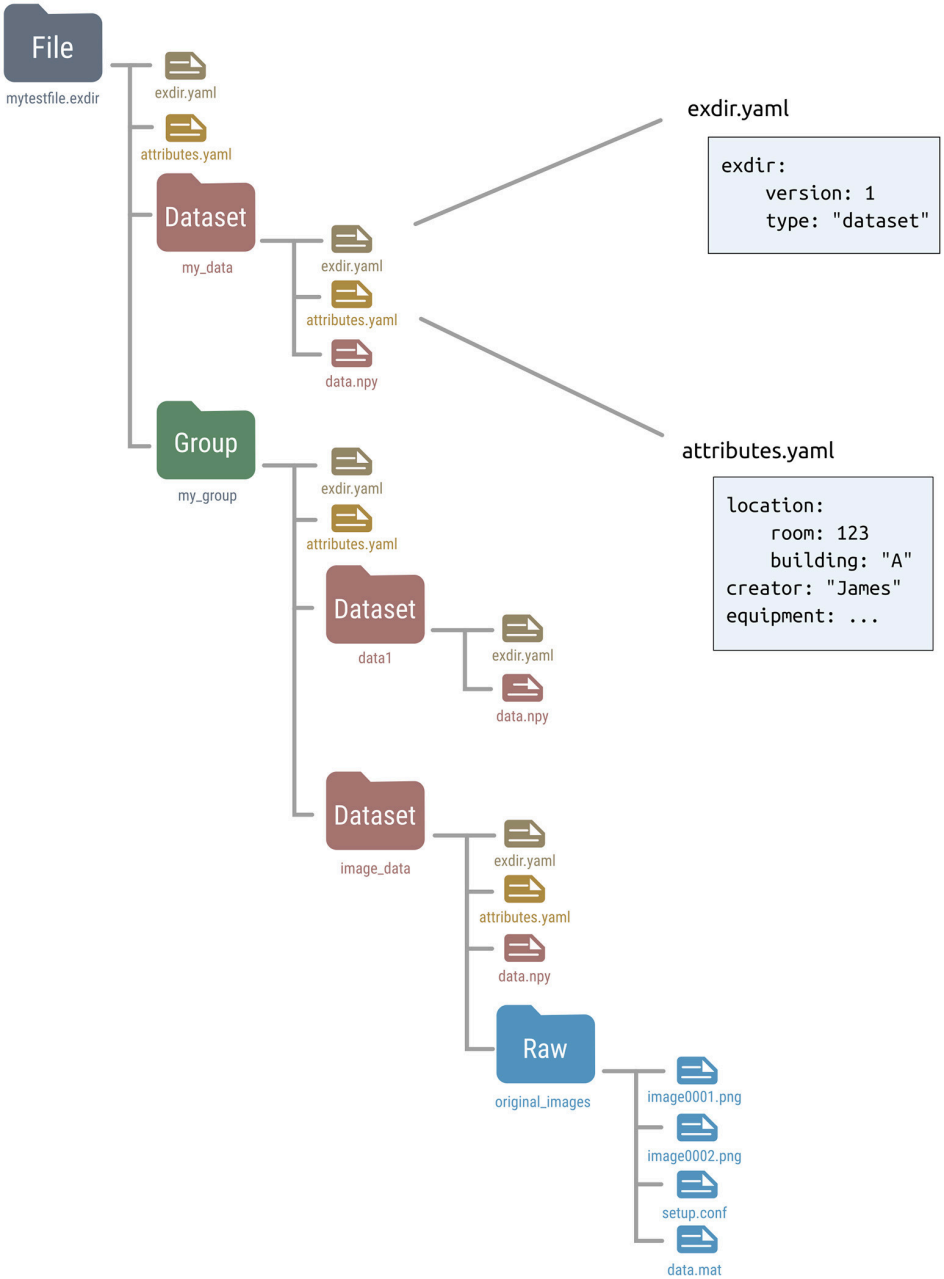
Overview of an example Exdir directory. File, Group, and Dataset refer to objects in Exdir, and are stored as directories in the file system. These objects are equivalent to the same objects in the HDF5 abstract data model. Raw is specific to Exdir and is a regular directory containing arbitrary data files. Inside each directory, there is a file named exdir.yaml with information about the object type and Exdir version. Each object may contain an attributes.yaml file containing user-defined attributes. Inside the Dataset directory is a file named data.npy that contains the data of the dataset stored in the NumPy binary format.

Exdir has four types of objects, File, Group, Dataset, and Raw, where each is represented as a directory in the file system. Raw is a type of object that is not present in the original HDF5 abstract data model. Metadata for each of these objects is stored in a file named exdir.yaml. All objects can have attributes stored in an optional file named attributes.yaml. Figure 1 shows an example structure of an Exdir File, and a summary of specifications of the data format is shown in Table 2.

**Table 2:** Exdir format structure.

**Type**	**Description**	**Contains**	**Required**	**Optional**
File		Group		
	Root object	Raw	exdir.yaml	attributes.yaml
		Dataset		
Group		Group		
	Intermediate directory	Raw	exdir.yaml	attributes.yaml
		Dataset		
Dataset			exdir.yaml	
	Data			attributes.yaml
			data.npy	
Raw				exdir.yaml
	Arbitrary data files			
				attributes.yaml

### 4.1. Metadata, attributes, and data files

Metadata for each object is stored in the exdir.yaml file in the object's directory. This file defines that the current directory is an Exdir object, and contains information about the Exdir version and object type. For example, this is the exdir.yaml file of a dataset:


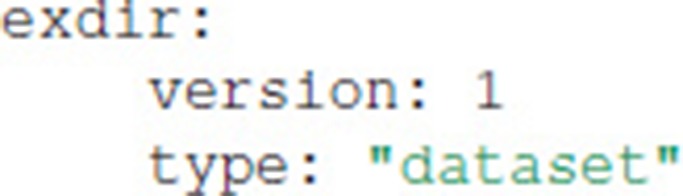


The object type can either be file, group, dataset, or raw. The exdir.yaml file is optional for Raw objects.

User-defined attributes of an Exdir object are stored in that object's directory in the attributes.yaml file. Attributes are stored as key–value pairs, which can be nested:


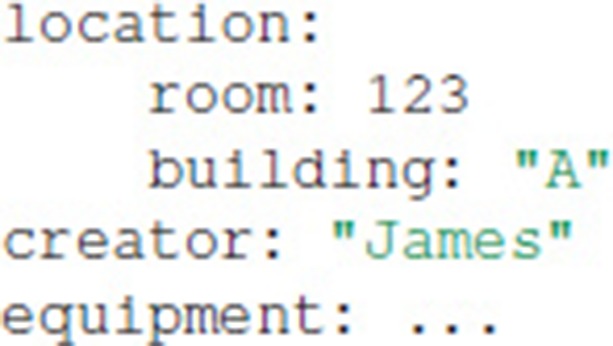


Binary data of a Dataset is stored in the NumPy format in a file named data.npy in the Dataset object's directory.

### 4.2. File, Group, and Dataset names

Because Exdir stores File, Group, and Dataset objects as directories in the file system, special care has to be taken to adhere to the different filename rules on major operating systems. While file and directory names are case-insensitive in Microsoft Windows, they are case-sensitive on most Linux file systems. If two datasets exist in the same directory with the same name, but different case, e.g., Name and name, then transferring the Exdir directory from a Linux system to a Windows system will result in a conflict.

Datasets and groups at the top level of any Group or File must have unique, case-insenitve names. However, Exdir is case-aware and case-preserving when quering and storing objects, which means that objects must be referenced with the exact case when queried by name.

### 4.3. File

The File object is the root (top level) object of an Exdir hierarchy. Every directory below a File in the directory hierarchy is part of that File. A File cannot contain other File objects. The metadata of the File is stored in exdir.yaml, and optional attributes in attributes.yaml.

### 4.4. Group

Inside the File, multiple objects may be stored, among them Group objects. Group objects may also contain any number of other Group objects, Raw objects, and Dataset objects. Group objects are stored as directories in the file system with metadata stored in exdir.yaml, and optional attributes in attributes.yaml. File objects are a specialization of a Group object.

### 4.5. Dataset

Dataset objects are for storing data. Dataset objects are stored as directories with metadata in the exdir.yaml file, and user-defined attributes in an optional attributes.yaml file. The data within a Dataset is stored in a binary NumPy file named data.npy, and thereby follows the specifications of the NumPy format.

### 4.6. Raw

Raw objects are used to store data in other formats than the NumPy format. While the user may store any type of data in the a Raw directory it is encouraged to use Dataset objects if possible. For Raw directories the exdir.yaml file is optional. Further, attributes are stored in the optional attributes.yaml file. There is no similar concept to Raw objects in HDF5.

## 5. Reference implementation in python

We have created a reference implementation of the Exdir specification in Python. This implementation is hosted on Github and is publicly available with an open-source license. It can easily be installed with Anaconda^16^.

The reference implementation of Exdir owes its relative simplicity to being based on existing formats, and to having a hierarchy based on regular file system directories. It is implemented using the open-source NumPy and PyYAML^17^ libraries, and is designed to be compatible with the popular HDF5 library, h5py. The compatibility should ease the transition from h5py to Exdir.

**Figure 2:**
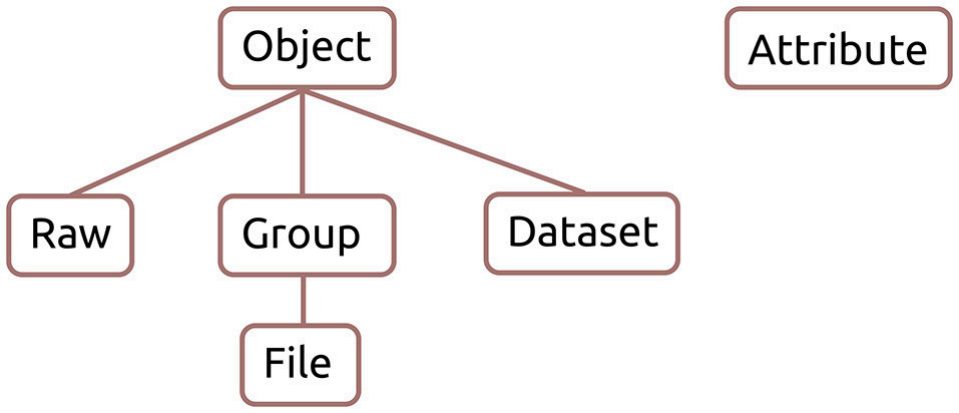
Exdir reference implementation class hierarchy.

The class hierarchy of the reference implementation is shown in Figure 2. The Raw, Group, and Dataset classes inherit from Object, which contains their common methods. The File class is a subclass of Group and they share many of the same methods. Attribute is a separate class that handles attributes for all Exdir objects. Furthermore, the reference implementation has an extensive test suite that can be run with pytest^18^.

### 5.1. Overview of the exdir API in python

In this section we give a quick overview of the Exdir Python API. An Exdir File is created as follows:


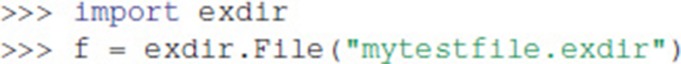


The File object points to the root directory in the Exdir directory structure. To create a Dataset inside the root directory (or other Group objects) the create_dataset() method can be used:


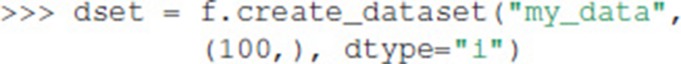


Exdir Dataset objects are not NumPy arrays, but behave similarly. They have both a shape and a data type:


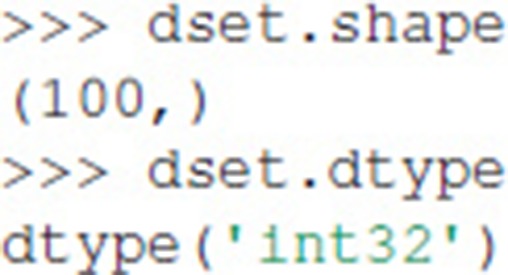


Dataset objects support array-style slicing, which can be used to read and write data to the Dataset:


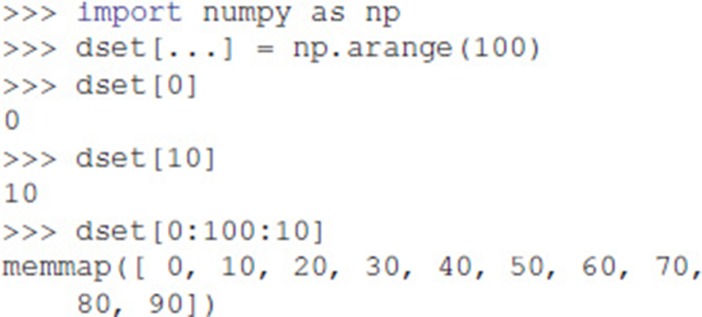


In addition, Dataset objects can also be created from the data directly:


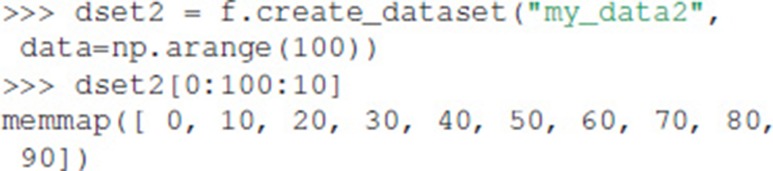


Exdir uses NumPy's memory mapping feature (memmap) to access segments of larger datasets on disk, without reading the entire file into memory. Furthermore, Exdir supports all the operations supported by memmap, including fancy indexing:


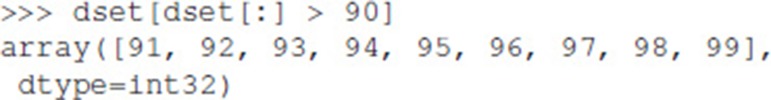


An Exdir Group can be created using create_group():


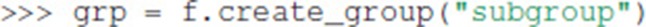


As with File objects, a Dataset is created inside a Group by using the create_dataset() method:


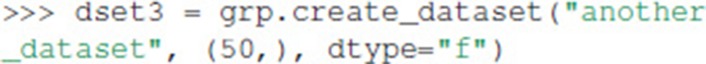


Group objects support most of the Python dictionary-style interface. You retrieve objects in the file using the item-retrieval syntax:


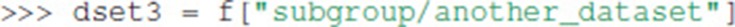


As shown above the name of objects follows the hierarchy of the POSIX standard with /-separators. To retrieve the name of any object in an Exdir directory one can use:


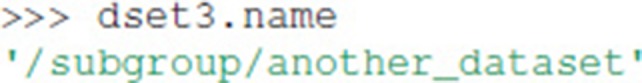


Iterating over a File or a Group provides the names of their members:


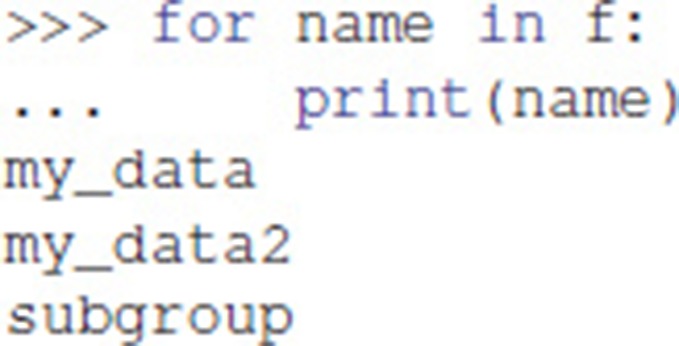


Containership testing also uses names:


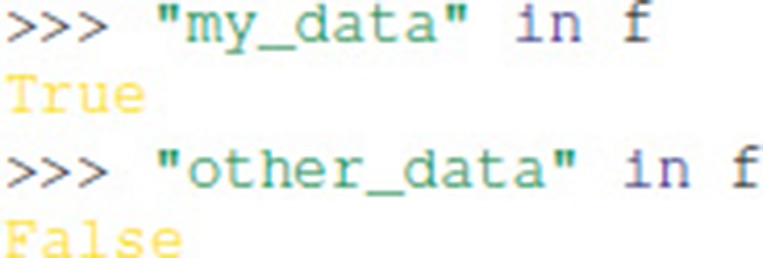


Group objects have the methods: keys(), values(), items(), iter(), and get().

All File objects, Group objects, and Dataset objects can have attributes. Attributes are accessed through the attrs property, which implements a dictionary interface:


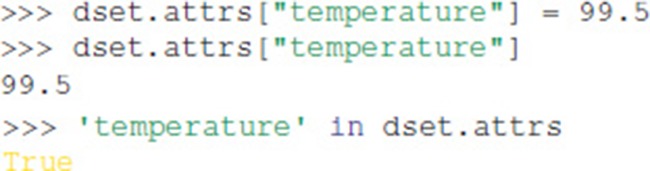


Unlike HDF5 and h5py, Exdir supports dictionaries as attributes:


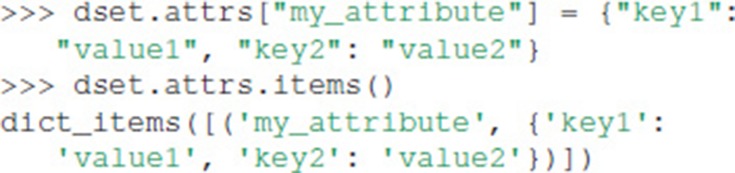


After the above commands, the Exdir directory structure becomes:


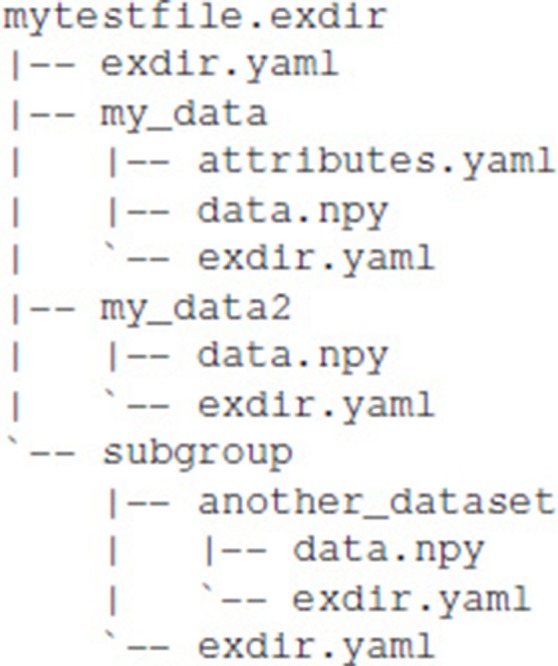


### 5.2. Exdir plugins

The functionality of Exdir can be extended with plugins. These allow modifying the behavior of Exdir when enabled. For instance, dataset and attribute plugins can perform pre- and post-processing of data during reading and writing operations. Note that plugins do not change the underlying specifications of Exdir. Plugins are intended to perform verification of data consistency, and to provide convenient mapping from general in-memory objects to objects that can be stored in the Exdir format and back again. Some plugins are provided in the exdir.plugins module, while new plugins can be defined by Exdir users or package developers.

One of the built-in plugins provides experimental support for units using the quantities package (Dale, 2017):


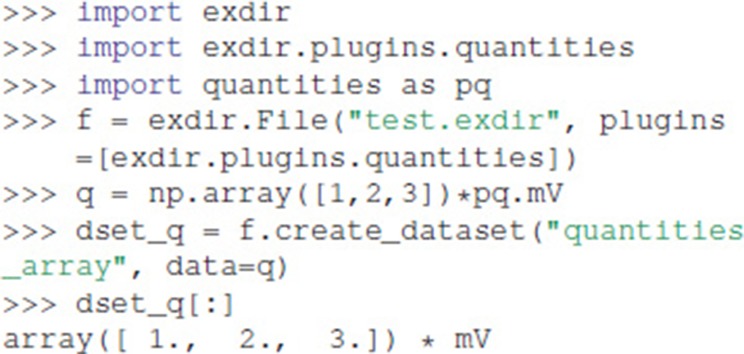


As shown in the above example, a plugin is enabled when creating a File object by passing the plugin to the plugin argument.

To create a custom plugin, one of the handler classes in exdir.plugin_interface must be inherited. The abstract handler classes are named after the object type you want to create a handler for. In this example we have a simplified Quantity class, which only contains a magnitude and a corresponding unit:


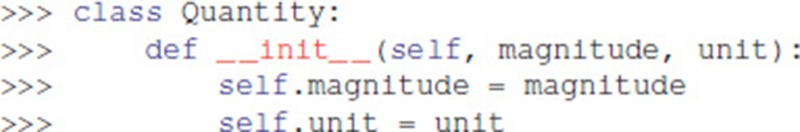


Below, we create a plugin that enables us to directly use a Quantity object as a Dataset in Exdir. We do this by inheriting from exdir.plugin_interface.Dataset and overloading prepare_write and prepare_read:


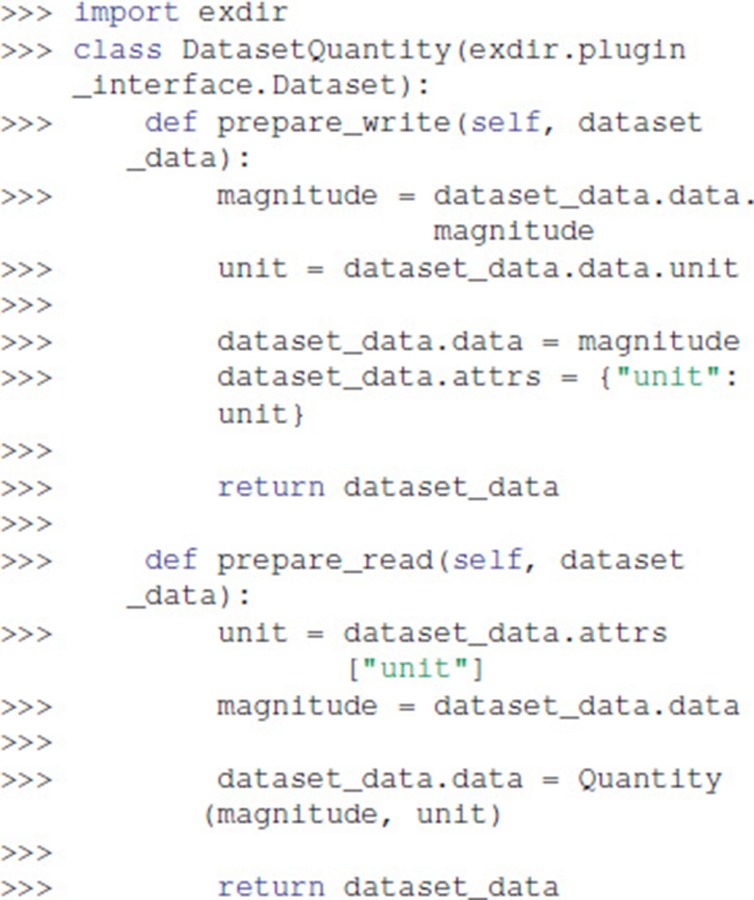


The overloaded functions take dataset_data as an argument. This has the data, attrs, and meta properties. The property attrs is a dictionary with optional attributes, while meta is a dictionary with information about the plugin. In prepare_write, the magnitude and unit of the data is translated to a value (numeric or numpy.ndarray) and an attribute (dictionary-like) that then can be written to file. prepare_read receives the data from the NumPy file and the attributes from the YAML file, and uses these to reconstruct a Quantity object.

We create a plugin that uses this handler as follows:


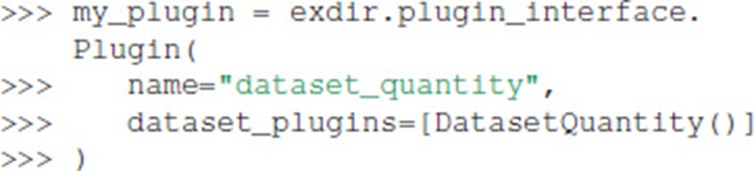


The plugin is enabled when opening a File by passing it to the plugins parameter:


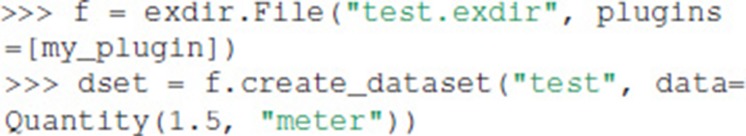


### 5.3. Converting from using HDF5 to exdir

As can be seen from Table 1, many common formats in neuroscience are based on HDF5. Since Exdir follows the abstract data model of HDF5, it is easy to switch from HDF5 to Exdir, and these formats should be able to support both HDF5 and exdir as backends. Often, the only change needed to transition from h5py to Exdir will be to switch from:


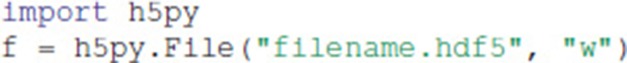


To the following:


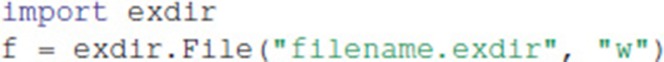


In most cases, the rest of the code can be left unchanged.

A few operators in h5py are missing in the reference implementation and will eventually be added. Furthermore, HDF5 has support for linking of objects, which is currently not part of the Exdir specification and will be added in the future. Finally, the reference implementation currently does not support parallel read/write operations on single stobjects. A future plugin is planned to provide such support.

### 5.4. Reading and writing to exdir in other languages

It is simple to load and edit Exdir objects in languages with existing NumPy and YAML libraries, such as in MATLAB. Here we show how to read Exdir objects with MATLAB after writing them with Python.

Assume that we have written an Exdir file with the following Python script:


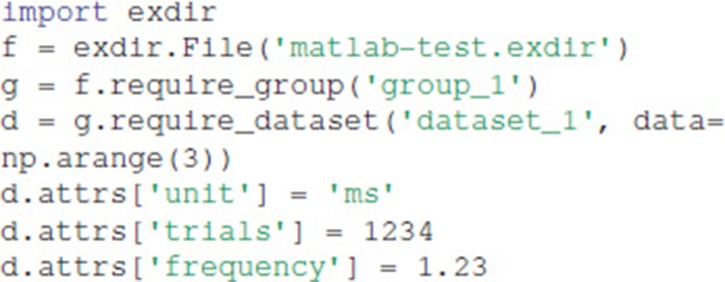


Then, in order to load the dataset as a vector with its corresponding attributes as a struct, one first has to add the path to npy-matlab^19^ and yamlmatlab^20^ with


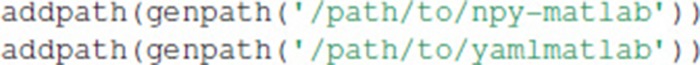


The data can be loaded into memory with the following code:


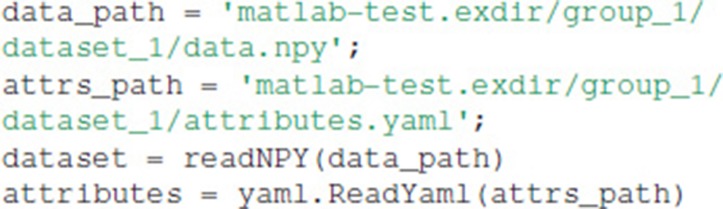


This results in the following output:


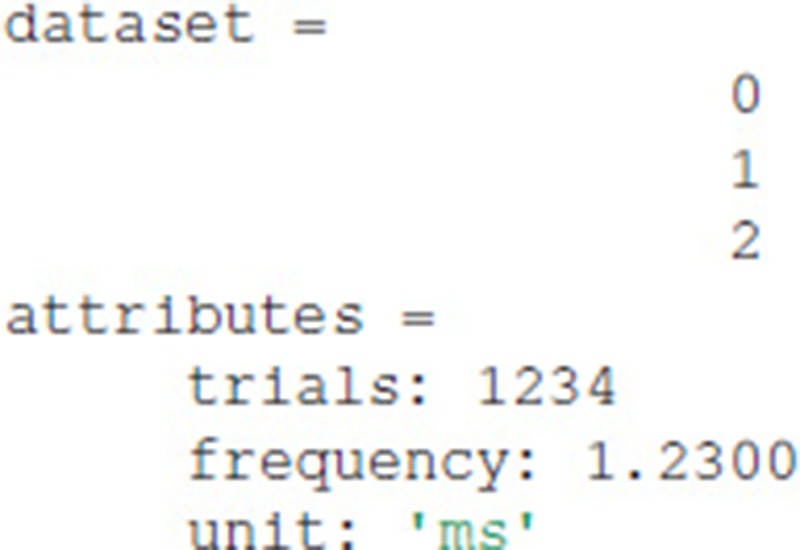


Editing the dataset and attributes is similarly easy:


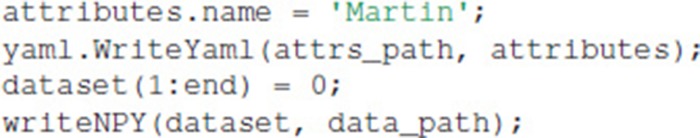


## 6. Tools for exdir

The Exdir command line interface and the Exdirare tools created to make it easier to work with Exdir data.

### 6.1. Exdir command line interface

Exdir-cli is a simple command line interface for browsing Exdir directories and to create Exdir File objects and Group objects. Listing the content of an Exdir File is done in the command line by the following:


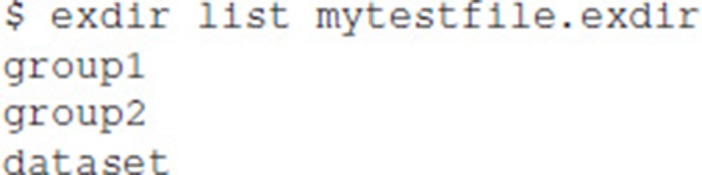


Listing the contents of a Dataset is done by the following:


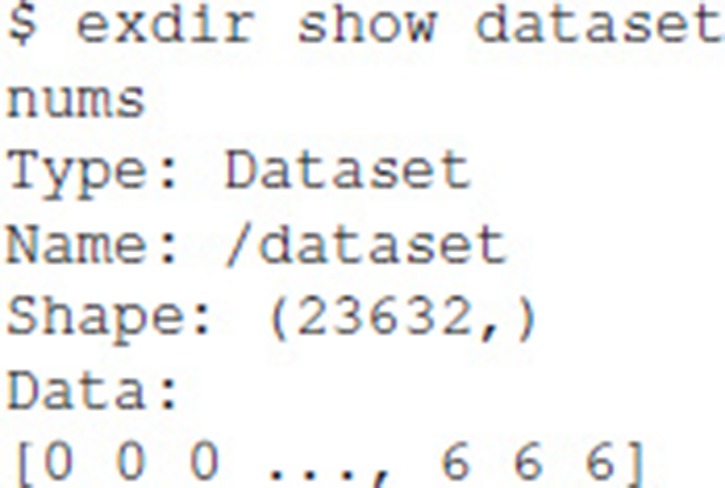


### 6.2. Exdir

**Figure 3:**
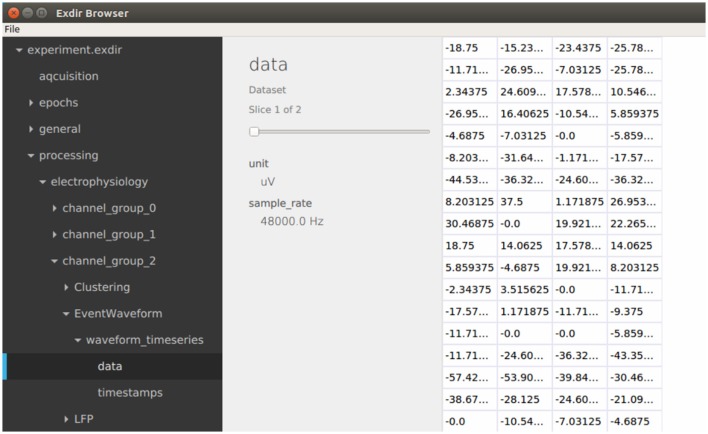
Screenshot of the Exdir browser.

Exdiris a graphical user interface for viewing and editing Exdir directories written in C++ using the open-source Qt application framework^21^ (see Figure 3). The browser can be installed on Linux, macOS, and Windows through Anaconda^22^ or from source^23^.

After opening an Exdir directory, the Exdirshows a hierarchical tree of all the objects in that directory. Information about each object is shown when selected and attributes of all objects may be edited. Group objects can be expanded to show their child objects, similar to directories on the file system. When selecting a dataset, the contents is shown in a 2D table. If the dataset is three dimensional, you can select the slice.

### 6.3. HDF5/exdir converter

In order to allow users to convert existing HDF5 files to Exdir, or the other way around, we have created a simple conversion tool^24^ which can be used from the command line. Converting from HDF5 to Exdir is done using hdf2exdir:


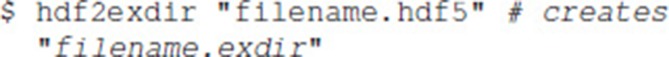


And converting from Exdir to HDF5 is done using exdir2hdf:


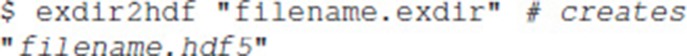


This converter is currently in development, but the main functionality is already implemented, including converting Group, Dataset, and Attribute objects.

## 7. Performance

As with other formats, the performance of Exdir is limited by the file system and underlying hardware. In general, data readability has been prioritized over performance in Exdir, but we are improving the performance where possible.

We have performed benchmarks for some common operations and compared the Exdir reference implementation to the h5py Python library. The benchmarks can be explored as a Jupyter notebook in the source code repository (see README.md for details) or online using Binder^25^. This notebook also contains examples that illustrate how the individual benchmarks can be profiled to identify performance bottlenecks.

The results are listed in Table 3. These results were found by performing the benchmarks on a desktop computer running Linux and a laptop computer running Microsoft Windows (see the table caption for details).

**Table 3:** Results from benchmarks comparing performance in Exdir with h5py.

**Library**	**h5py**	**Exdir**	**h5py**	**Exdir**
**OS**	**Linux**	**Linux**	**Windows**	**Windows**
Add 5 attributes	0.002 s	0.010 s	0.002 s	0.030 s
Add 200 attributes (one by one)	0.066 s	3.6 s	0.068 s	5.5 s
Add 200 attributes (single operation)	N/A	0.030 s	N/A	0.049 s
Add dataset with 10^6^ 64-bit floats	0.009 s	0.013 s	0.019 s	0.040 s
Add dataset with 10^8^ 64-bit floats	0.38 s	0.54 s	4.4 s	0.83 s
Create 5,000 groups (thorough validation)	0.26 s	8.1 s	0.36 s	8.9 s
Create 5,000 groups (minimal validation)	0.26 s	1.1 s	0.36 s	8.4 s
Create tree (3 groups × 5 levels)	0.14 s	0.34 s	0.14 s	2.1 s
Write 3D slice (100 × 300 × 100)	0.0033 s	0.0048 s	0.031 s	0.029 s

*A 2GB RAM disk was used as virtual hard drive for the tests. Filename validation was disabled for Exdir in all tests. Software used: Python 3.6, NumPy 1.13.1, Ubuntu 16.04, Windows 7. Hardware used: Linux: Intel Core i7-5820K 3.30 GHz, 32 GB RAM, Intel 535 512 GB SSD. Windows: HP EliteBook 8570p, Intel Core i7-3520M, 2.90 GHz, 8 GB RAM. Samsung 128 GB SSD*.

As can be seen from “Add 200 attributes (one by one)” in Table 3, adding 200 attributes one by one is slow in Exdir compared to h5py. This is because each written attribute results in a complete rewrite of the “attributes.yaml” file. The performance might be improved by caching the changes and flushing them to the file at regular intervals, but we have chosen to postpone the addition of such features to keep the current implementation simple. However, as is shown in “Add 200 attributes (single operation)” in Table 3, it is possible to emulate this behavior by first adding the same attributes to a Python dictionary and then assign them to the attrs property of an object. Adding many attributes in a single operation with Exdir is faster than adding them one by one with h5py. It should be noted that is only possible in Exdir, and not supported by h5py.

Further, manipulation of metadata in Exdir has an added benefit over HDF5 on networked file systems if the file system downloads and uploads entire files when they are modified. Metadata in Exdir is stored in separate files, and only these files need to be downloaded, while the rest of the dataset can remain on the server. This is in contrast to HDF5 where the entire file may have to be downloaded.

Reading and writing large continuous data in Exdir is about as fast as with h5py on Linux, and faster on Windows. This is also the case for reading and writing to parts of a dataset. However, HDF5 supports storing chunked data, which is a feature missing in Exdir, and in these cases, HDF5 is likely to outperform Exdir when reading and writing binary data.

Creating many empty objects is slower with Exdir than with h5py, as shown in the “Create 5000 groups (thorough validation)” benchmark in Table 3. Profiling this example on Linux shows that most of the time in Exdir is spent on filename validation. In the reference implementation, “thorough” validation is enabled by default and will enforce the Exdir naming rules discussed in section 4.2 by checking if an existing object exists in the same folder with the same name but different case. This is to avoid name conflicts when directories created on case-sensitive operating systems are transferred to case-insensitive operating systems. This check is very time-consuming when many objects are present, and can be disabled by choosing a different validation function. For instance, “minimal” validation can be enabled by passing name_validation=exdir.validation.minimal to the File constructor. Minimal validation only checks if a file or folder with the exact, case-sensitive name already exists. This is much faster on Linux and brings the performance of Exdir closer to that of h5py, as shown in the “Create 5000 groups (minimal validation)” benchmark in Table 3. With minimal validation, Exdir spends most time on creating directories and the exdir.yaml files. On Windows, thorough name validation does not explicitly compare the names by iterating over all existing files and directories because this is already done by default by the operating system's check for file existence. However, the performance on Windows is almost as bad as with thorough validation enabled on Linux. Profiling this benchmark on Windows shows that much of the time is spent on on low-level file system operations, such as nt.open, nt.mkdir, and nt.stat. It therefore seems unlikely that performance can be improved much in this case.

In summary, the performance of Exdir is mostly limited by the performance of the file system and the performance of the YAML and NumPy libraries. Exdir performs worse than h5py with many individual operations on attributes, but performs better if the individual operations are accumulated into a single operation. Exdir performs worse than h5py with many small objects, which means that HDF5 is a better alternative for use cases where many small objects need to be written with high performance. However, when writing large datasets, Exdir performs similarly or better than h5py.

## 8. Discussion

We have proposed a new specification, Exdir, that puts the abstractions of HDF5 on top of a hierarchical directory structure. Exdir gives the same flexibility as HDF5, but with the advantages of a simpler specification, human-readable metadata, and applicability of established tools. Further, the hierarchy and metadata can be modified manually without tools specific to Exdir, while the data is accessible by existing libraries for common languages. This makes Exdir a possible replacement for HDF5 in computational and experimental data pipelines.

We have presented a reference implementation in Python, a command-line client, and a graphical browser that are all open source and available on GitHub. Together, these tools will hopefully make it easy for other researchers to explore the specification and provide valuable feedback. Because Exdir is based on the established NumPy and YAML formats, we expect APIs for other languages to be fairly easy to implement.

The reference implementation has an extensive test suite and has been thoroughly tested, although the format is still under development. The flexibility of the format gives many possibilities for future development. Exdir includes the concept of plugins, which makes it easy to extend implementations with new functionality without adding more complexity to the specification.

Because similar strategies for data storage are already in use, but no formal standard exists, we believe Exdir provides an opportunity for increased data sharing and development of tools that can be shared across multiple disciplines. We hope Exdir can lay the foundation for a standardization of such strategies, and contribute to the general discussion on data storage in science.

## Author contributions

S-AD, MH, and ML conceived of and designed the project; S-AD, MH, ML, and ST wrote software, documentation, and the paper. All authors contributed to revising the paper and approved of the final version.

### Conflict of interest statement

S-AD is employed part-time by The Qt Company. The Qt Company develops the Qt application framework, which the authors used to create the Exdir Browser. The other authors declare that the research was conducted in the absence of any commercial or financial relationships that could be construed as a potential conflict of interest.

## Footnotes

^1^https://support.hdfgroup.org/HDF5/doc/

^2^https://github.com/CINPLA/exdir/

^3^http://git-scm.org

^4^https://www.gnu.org/software/wdiff/manual/wdiff.html

^5^http://meldmerge.org/

^6^https://support.hdfgroup.org/HDF5/docNewFeatures/FileSpace/RFC-Paged_Aggregation.pdf

^7^https://support.hdfgroup.org/HDF5/users5.html

^8^http://yaml.org/

^9^https://github.com/ewiger/yamlmatlab

^10^yaml.org/spec/1.2/spec.html

^11^https://docs.scipy.org/doc/numpy/neps/npy-format.html

^12^https://github.com/kwikteam/npy-matlab

^13^https://github.com/rogersce/cnpy

^14^https://git-scm.com

^15^https://git-annex.branchable.com/

^16^https://anaconda.org/cinpla/exdir

^17^http://pyyaml.org

^18^https://docs.pytest.org

^19^https://github.com/kwikteam/npy-matlab

^20^https://github.com/ewiger/yamlmatlab

^21^https://www.qt.io/

^22^https://anaconda.org/cinpla/exdir-browser

^23^https://github.com/CINPLA/exdir-browser

^24^https://github.com/CINPLA/hdf5-exdir-converter

^25^https://mybinder.org/v2/gh/CINPLA/exdir/dev?filepath=tests/benchmarks/benchmarks.ipynb
